# Supplementary material for: MetaRibo-Seq measures translation in microbiomes
Source: Nat Commun. 2020 Jun 29;11:3268. doi: 10.1038/s41467-020-17081-z (PMC7324362; doi:10.1038/s41467-020-17081-z)
Supplement: Supplementary file 10 — Supplementary Data 7 [file 41467_2020_17081_MOESM10_ESM.zip › File2/Confidence_High_Taxonomy/216672_out.krona.html]

Javascript must be enabled to view this page.

members
magnitude
magnitudeUnassigned
count
unassigned
taxon
rank

216672\_out

2

superkingdom
2
2

phylum
1
1239

186801
1
class

1
186802
order

family
186803
1

genus
33042
1

SRS142923\_contig\_number\_contig-100\_25263.25264

phylum
1224
1

1
1236
class

1
135625
order

1
712
family

724
1
genus

726
1
species

SRS064809\_contig\_number\_contig-100\_4923.27631
